# A Functional TGF-β/Smad Assay for Targeted Profiling of Demineralized Bone Matrix-Derived Allograft Bioactivity

**DOI:** 10.3390/jfb17080416

**Published:** 2026-08-19

**Authors:** Adrian Lendvai, Tobias Weichhart, Hans Peter Weitzenböck, Christoph Wiesner, Rita Seeboeck, Narges Zamani, Michael Matzner, Monika Pichler, Bettina Steiner, Andrea De Luna, Stefan Nehrer, Harald Hundsberger

**Affiliations:** 1 Department of Life Sciences, IMC University of Applied Sciences, 3500 Krems, Austria; adrian.lendvai@imc.ac.at (A.L.); hans.weitzenboeck@imc.ac.at (H.P.W.); christoph.wiesner@imc.ac.at (C.W.); rita.seeboeck@imc.ac.at (R.S.);; 2 Center for Regenerative Medicine, University for Continuing Education Krems, 3500 Krems, Austria; andrea.de-luna@donau-uni.ac.at (A.D.L.); stefan.nehrer@donau-uni.ac.at (S.N.); 3 Institute of Clinical Pathology and Molecular Pathology of the Lower Austria Central Region (University Hospital St. Pölten), Karl Landsteiner University of Health Sciences, 3500 Krems, Austria; 4 Cells + Tissuebank Austria gemeinnützige GmbH, 3500 Krems, Austria; michael.matzner@meduniwien.ac.at (M.M.); m.pichler@ctba.at (M.P.); b.steiner@ctba.at (B.S.); 5 Department of Orthopedics and Traumatology, Medical University of Vienna, 1090 Vienna, Austria; 6 Department of Dermatology, University Hospital of the Paracelsus Medical University, 90419 Nürnberg, Germany

**Keywords:** demineralized bone matrix, extracellular matrix, TGF-β, HEK-Blue reporter assay, allograft, bone regeneration, functional bioactivity, biomaterial screening

## Abstract

Demineralized bone matrix (DBM)-derived allografts retain extracellular matrix (ECM)-associated factors involved in bone repair, but biochemical protein recovery alone may not predict functional pathway activation. We evaluated the HEK-Blue™ TGF-β Reporter Assay as a targeted method for assessing Smad-dependent reporter activity in DBM-derived materials. ECM proteins were extracted from cortical demineralized bone granules (DBG) using guanidine hydrochloride (GuHCl) or urea and quantified after extraction, dialysis, and sterile filtration. Reporter cells were stimulated with extracted ECM proteins or directly with processed cortical and cancellous products, including DBG, wet heat-treated DBG formulated as Putty (PHT), and gamma-irradiated PHT (PGI). Non-pooled DBM sponge samples were also tested. Secreted embryonic alkaline phosphatase (SEAP) activity served as the functional reporter readout. Material-only no-cell controls assessed material-derived background. PrestoBlue™ readouts served as exploratory quality controls. The urea-derived extract yielded a higher apparent BCA-detectable protein concentration than the GuHCl-derived extract. Only the GuHCl-derived extract induced increasing SEAP activity at matched protein input. Direct stimulation showed reporter activation above the TNF-α pathway-negative cytokine control for cortical DBG, PHT, PGI, and cancellous DBG. DBM sponges also induced detectable SEAP activity. These findings support targeted functional screening of Smad-dependent TGF-β reporter activation in DBM-derived materials, but not total osteoinductivity assessment.

## 1. Introduction

Bone graft-derived materials are widely used to support bone repair when endogenous healing capacity is insufficient. Although autologous bone remains biologically attractive as it combines osteoconductive, osteoinductive, and osteogenic properties, its clinical use is limited by donor-site morbidity, restricted availability, and variable graft quality [1,2,3,4,5,6]. Demineralized bone matrix (DBM)-derived allografts are therefore widely used alternatives, as demineralization exposes the organic extracellular matrix and may retain matrix-associated factors involved in bone regeneration [7,8].

However, the functional assessment of DBM-derived materials remains challenging. DBM-associated activity can be influenced by donor-related variables, anatomical source, demineralization, chemical processing, sterilization, storage, carrier formulation, and final product format [9,10,11,12,13,14,15,16]. Accordingly, DBM-derived products are supplied in several formats, including granules, fibers, sponges, putties, pastes, and carrier-based formulations [8,11,12,13]. These formats differ not only in handling properties but also in material geometry, hydration behavior, matrix presentation, and carrier-associated composition, all of which may influence how matrix-associated factors are retained, released, or presented to cells. Conventional biochemical characterization, including total protein quantification or detection of growth-factor-related epitopes, provides useful information on extractable material components but does not demonstrate whether these components activate cellular signaling pathways [17,18,19,20,21].

Functional cell-based assays can address this gap by measuring pathway activation rather than bulk protein recovery alone. Extraction-based workflows allow soluble extracellular matrix (ECM) fractions to be tested at defined protein inputs, whereas direct material testing preserves aspects of the supplied product state, including geometry, hydration, matrix-bound components, and surface-associated cues [22,23,24,25,26,27,28,29]. These approaches provide complementary information on extractable and material-state-dependent bioactivity. Among bone-associated signaling systems, TGF-β/Smad signaling represents a relevant pathway-specific readout as TGF-β is stored in the bone matrix and contributes to bone remodeling, osteoprogenitor recruitment, osteoblast and osteoclast regulation, and ECM homeostasis [30,31,32,33,34,35,36]. Functional TGF-β activity has been detected in DBM-derived preparations, supporting the use of reporter-cell systems to assess whether matrix-associated factors can activate TGF-β/Smad signaling [37,38,39,40]. Nevertheless, TGF-β/Smad activity reflects one functional component of DBM-associated biology and may serve as a candidate readout for assessing selected aspects of DBM-associated osteoinductive or regenerative potential, while not capturing overall graft bioactivity.

In this study, we evaluated the HEK-Blue™ TGF-β Reporter Assay as a targeted functional screening approach for DBM-derived allograft materials. The assay was performed on ECM extracts derived from cortical DBG, directly processed cortical and cancellous allograft products, and donor-specific DBM sponge samples. SEAP activity served as the Smad-dependent reporter readout. Material-only no-cell controls were included for direct DBG, PHT, and PGI stimulation, and PrestoBlue™ readouts were used as assay-control measurements. This design allowed us to compare extractable and material-state-dependent TGF-β/Smad reporter activity and to define methodological considerations for functional screening of DBM-derived biomaterials. As HEK-Blue™ TGF-β reporter cells express SEAP under the control of a Smad-inducible promoter, the assay provides a pathway-specific readout of TGF-β/Smad signaling rather than a general measure of cellular activity. As receptor-level confirmation by pathway inhibition or TGF-β neutralization was not included, the findings are interpreted as Smad-inducible reporter activity in a TGF-β-responsive reporter system rather than as pharmacologically confirmed TGF-β receptor-dependent activation.

## 2. Materials and Methods

### 2.1. Bone Graft Preparation

Cortical and cancellous allograft materials were supplied by the Cells + Tissuebank Austria gemeinnützige GmbH, Krems, Austria (Austrian commercial register no. FN 243394h, Regional Court Krems). Cortical samples originated from femoral diaphyses, whereas cancellous samples were obtained from femoral heads. Native bone was processed using the Allotec^®^ purification procedure under the tissue bank’s internal regulatory framework. The workflow included: mechanical cleaning by cutting, sawing, and centrifugation, chemical cleaning and subsequent lyophilization, demineralization, heat treatment, rehydration, Putty formulation, and sterilization. Demineralization was performed with 0.6 M HCl, and thermal treatment was conducted below 65 °C. Details of heat treatment, rehydration, and Putty formulation are not disclosed as these steps are part of the tissue bank’s validated manufacturing process. Processing was performed under the tissue bank’s internal regulatory framework and in accordance with applicable national and international requirements, including EDQM guidance and the Austrian Gewebebankenverordnung [41,42]. These steps generate paste-like, moldable graft preparations. Final sterilization was performed by gamma irradiation at 25–35 kGy in accordance with the manufacturer’s validated protocol.

Three processing stages were investigated: demineralized bone granules (DBG), wet heat-treated DBG formulated as Putty (PHT), and gamma-irradiated PHT (PGI). Each stage was represented by cortical and cancellous products, yielding six processed-material conditions. These products were derived from pooled manufacturing material, with donor-pool sizes varying by processing stage and material type. Consequently, the DBG, PHT, and PGI samples represent pooled product-level materials rather than donor-level biological replicates. Independent replicates in the reporter assays refer to separate cell-culture experiments and, where applicable, independent product batches. Technical replicates were performed in triplicate. Exact donor-pool sizes, batch identifiers, and batch compositions are not disclosed to protect proprietary manufacturing information and anonymized donor-pool data. Donor-level variables, including sex and age, were therefore unavailable for stratified analysis. Two additional donor-specific DBM sponge samples, DBM 1 and DBM 2, were analyzed separately without pooling or homogenization.

### 2.2. ECM Extraction, Processing, and Protein Quantification from Cortical DBG

Extracellular matrix proteins were extracted from cortical DBG using one workflow per buffer condition. For each buffer condition, 25 mg cortical DBG were incubated in 500 µL extraction buffer containing either 4 M urea (Carl Roth GmbH + Co. KG; Karlsruhe, Germany; 3941.3) or 4 M guanidine hydrochloride (GuHCl) (Sigma-Aldrich; Taufkirchen, Germany; G3272-100G) in 20 mM ammonium bicarbonate (ABC) buffer, pH 8.0 (Merck KGaA; Darmstadt, Germany; 09830-500G). Extraction was performed for 24 h at room temperature under constant agitation. Lysates were clarified by centrifugation at 8000× *g* for 1 min, followed by 12,000× *g* for 1 min. Supernatants were dialyzed overnight against 850 mL of 1× PBS using dialysis membrane tubing with a molecular weight cut-off (MWCO) of 1 kDa (Spectrum Chemical; New Brunswick, NJ, USA; 132103) and sterile-filtered through 0.22 µm filters (VWR; Vienna, Austria; 514-1251P). Residual urea or GuHCl concentrations after dialysis were not analytically quantified, and extraction-buffer-only blanks dialyzed in parallel were not included in the BCA workflow. Preliminary compatibility testing did not indicate overt incompatibility of the dialyzed preparations with reporter-cell stimulation under the applied assay conditions. However, residual-buffer effects on BCA quantification or reporter activity cannot be excluded. Protein concentrations are therefore reported as apparent BCA-detectable protein concentrations.

Apparent BCA-detectable protein concentrations were determined using the BCA Protein Assay Kit according to the manufacturer’s instructions (Cell Signaling Technology Europe; Leiden, The Netherlands; 7780S). A bovine serum albumin (BSA) standard curve ranging from 0 to 0.5 µg/µL in six dilutions with 0.1 µg/µL increments was used. Prior to BCA-based protein quantification, ECM extract samples were diluted 1:10 in 1× PBS, and protein concentrations were calculated by accounting for the dilution factor. To characterize the ECM preparation workflow, protein concentrations were measured after extraction, after dialysis, and after sterile filtration.

### 2.3. HEK-Blue™ TGF-β Reporter Cell Culture

HEK-Blue™ TGF-β reporter HEK-293 cells were used to assess TGF-β/Smad pathway activation (InvivoGen; San Diego, CA, USA; hkb-tgfbv2). Upon pathway activation, the cells express secreted embryonic alkaline phosphatase (SEAP) under the control of a Smad-inducible promoter. Thus, the assay reports Smad-inducible activation in a TGF-β-responsive reporter cell system rather than direct quantification of TGF-β protein concentration. Cells were cultured in high-glucose Dulbecco’s modified Eagle medium (DMEM) with pyruvate (Thermo Fisher Scientific; Vienna, Austria; 41966029), supplemented with 10% fetal bovine serum (FBS) (Thermo Fisher Scientific; Vienna, Austria; A5256701), 90 U/mL penicillin, 90 µg/mL streptomycin (Thermo Fisher Scientific; Vienna, Austria; 15140122), 100 µg/mL Normocin^®^ (InvivoGen; San Diego, CA, USA; ant-nr-05), and 100 µg/mL Zeocin^®^ (InvivoGen; San Diego, CA, USA; ant-zn-05). Cells were maintained at 37 °C and 5% CO_2_ and used at passages 5–7. Cell handling, stimulation, and test medium preparation were performed according to the manufacturer’s protocol.

### 2.4. Experimental Design of the HEK-Blue™ TGF-β Reporter Assay

The HEK-Blue™ TGF-β Reporter Assay was used to assess activation of the TGF-β/Smad pathway after stimulation with ECM extracts or with allograft materials applied directly. Unless otherwise stated, reporter cells were seeded at 50,000 cells per well and stimulated for 48 h at 37 °C and 5% CO_2_. Recombinant human TGF-β was used as a positive control at a final concentration of 1 ng/mL, while recombinant human TNF-α was used as a pathway-negative cytokine control at 10 ng/mL. TNF-α was included as the pathway-negative cytokine control according to the manufacturer’s HEK-Blue™ TGF-β reporter assay workflow, representing cytokine exposure that is not expected to activate the Smad-inducible reporter. This control was used as an operational negative reference for reporter activation. Untreated or vehicle-only wells were not included in the final statistical design. Stimuli were prepared in test medium according to the manufacturer’s instructions. All SEAP reporter assays were performed in three independent cell-culture experiments, with each condition analyzed in technical triplicates. For the ECM extract experiments, aliquots of the same single GuHCl-derived and urea-derived preparations were tested in the three independent cell-culture experiments. These experiments therefore represent independent assay runs but not independent extraction replicates. Material-only no-cell controls were included for direct DBG, PHT, and PGI stimulation to assess material-derived background signal. No-cell controls were not included for donor-specific DBM sponge samples.

### 2.5. ECM Extract Stimulation

For ECM extract stimulation, HEK-Blue™ TGF-β Reporter cells were treated with urea- or GuHCl-derived cortical DBG extracts. The GuHCl extract served as a reference for preparing the 20, 30, and 40 vol% treatment conditions in a final assay volume of 200 µL. Based on the measured GuHCl apparent BCA-detectable protein concentration, these conditions corresponded to 11.89, 17.84, and 23.78 µg apparent BCA-detectable ECM protein per well, respectively. As the urea extract contained higher apparent BCA-detectable protein concentrations, urea extract volumes were adjusted to match the corresponding GuHCl-derived apparent BCA-detectable protein amounts. Thus, urea and GuHCl extraction conditions were compared at equivalent apparent BCA-detectable ECM protein input. SEAP activity was quantified from culture supernatants, and PrestoBlue™-based viability/metabolic activity was assessed in parallel.

### 2.6. Direct Stimulation with Processed Allograft Products

For direct stimulation, approximately 10 mg of DBG, 20 mg of PHT, or 20 mg of PGI were added to each well of a 96-well plate and pre-wetted with 20 µL of test medium before cell seeding. For DBG, mass refers to the weighed granule material as supplied. For PHT and PGI, mass refers to the total supplied formulation, including the hydrated Putty product format, rather than dry DBM-equivalent mass. Material amounts were selected based on product format, handling feasibility, and compatibility with the 96-well assay format. They were not normalized to dry DBM content, ECM content, surface area, accessible matrix surface, hydration state, or dry-weight-equivalent input. Reporter cells were seeded directly onto the materials in 180 µL test medium. Supernatants were collected for SEAP quantification. PrestoBlue™ readouts for direct stimulation with DBG, PHT, and PGI were obtained from a single cell-culture experiment and evaluated descriptively as exploratory assay-quality control data due to potential material-associated assay interference. In this direct-contact format, cells and materials were physically co-localized within the same well. Separation and recovery of cells from particulate or Putty-like materials would introduce material-format-dependent detachment, recovery, and retention bias. Because no validated material-compatible normalization method was available for this assay format, the direct-contact SEAP readout was not normalized to cell number.

### 2.7. Donor-Specific DBM Sponge Stimulation

Two donor-specific DBM sponge samples, DBM 1 and DBM 2, were analyzed without pooling or homogenization. DBM sponges were cut to fit the well format with a thickness of approximately 1 mm. Reporter cells were seeded directly onto the sponge samples in 200 µL test medium and incubated under the same assay conditions described above. SEAP activity was quantified in culture supernatants. No-cell controls were not included because the original sponge experiment was designed as a feasibility assessment of the reporter workflow in structured DBM samples. Microscopic inspection was performed during the assay to monitor cell attachment and morphology.

### 2.8. SEAP Reporter Assay

SEAP activity was quantified using QUANTI-Blue™ according to the manufacturer’s instructions (InvivoGen; San Diego, CA, USA; rep-qbs). After stimulation, 20 µL culture supernatant was mixed with 180 µL QUANTI-Blue™ solution and incubated for 30 min at 37 °C. Optical density was measured at 630 nm. SEAP activity was reported as raw OD630 and evaluated relative to the TNF-α pathway-negative cytokine control. For direct-contact material stimulation, SEAP activity was reported as a non-normalized total well-level reporter output. The SEAP signal was interpreted as functional activation of the TGF-β/Smad reporter rather than as direct quantification of TGF-β protein concentration.

### 2.9. PrestoBlue™ Cell Metabolic Activity Assay

Cell metabolic activity was assessed using the PrestoBlue™ assay according to the manufacturer’s instructions (Invitrogen™; Carlsbad, CA, USA; A13261). After removal of 20 µL supernatant for SEAP analysis, 20 µL PrestoBlue™ reagent was added to the remaining 180 µL medium and incubated for 30 min at 37 °C and 5% CO_2_. Subsequently, 50 µL supernatant was transferred to a new plate, and fluorescence was measured at 560/590 nm. For ECM extract experiments, PrestoBlue™-based viability/metabolic activity was assessed in three independent cell-culture experiments with technical triplicates. For direct-contact stimulation with DBG, PHT, and PGI, PrestoBlue™ readouts were obtained in one of the three independent cell-culture experiments and were treated as exploratory assay-quality control data rather than as validated viability, proliferation, cytotoxicity, or cell-number endpoints. These data were not used to normalize SEAP activity.

### 2.10. Data Analysis and Statistics

Statistical analysis was performed using GraphPad Prism 11.0.1 (GraphPad Software; Boston, MA, USA). Technical triplicates were averaged before statistical analysis. For ECM extract stimulation and donor-specific DBM sponge stimulation, statistical analysis was performed on the means of three independent cell-culture experiments per condition. For direct stimulation with DBG, PHT, and PGI, technical triplicates were first averaged for each product batch within each cell-culture experiment. The batch means were then averaged within each cell-culture experiment, yielding three independent experiment means per material condition for statistical analysis. Recombinant TGF-β and TNF-α control conditions were analyzed as three independent experiment means after averaging technical triplicates. Statistical comparisons were performed against the TNF-α pathway-negative control using one-way ANOVA followed by Dunnett’s post hoc test. TNF-α was selected as the primary statistical reference as it served as the pathway-negative cytokine control in the HEK-Blue™ TGF-β reporter assay workflow. The statistical design was intended to identify reporter activation above this operational negative cytokine reference, not to establish process-to-process superiority among DBG, PHT, and PGI. No inferential pairwise comparisons were performed between DBG, PHT, and PGI or between cortical and cancellous material groups. Data are presented as mean ± SD. *p* ≤ 0.05 was considered statistically significant and indicated as follows: * *p* ≤ 0.05, ** *p* ≤ 0.01, *** *p* ≤ 0.001, **** *p* ≤ 0.0001.

## 3. Results

### 3.1. Overview of the Functional Reporter-Assay Workflow

The HEK-Blue™ TGF-β Reporter Assay was applied to DBM-derived allograft materials in three experimental settings. First, ECM extracts were prepared from cortical DBG using GuHCl or urea, and protein concentrations were measured after extraction, dialysis, and sterile filtration. Second, reporter cells were stimulated with defined protein inputs of GuHCl- or urea-derived ECM extracts. Third, reporter cells were stimulated directly with processed cortical and cancellous allograft products and with two donor-specific DBM sponge samples. SEAP activity was used as a reporter readout. Material-only no-cell controls were included to assess material-derived QUANTI-Blue™ background for direct DBG, PHT, and PGI stimulation, whereas PrestoBlue™ measurements served as metabolic assay-control readouts.

### 3.2. Protein Concentrations During ECM Extract Processing

**Figure 1:**
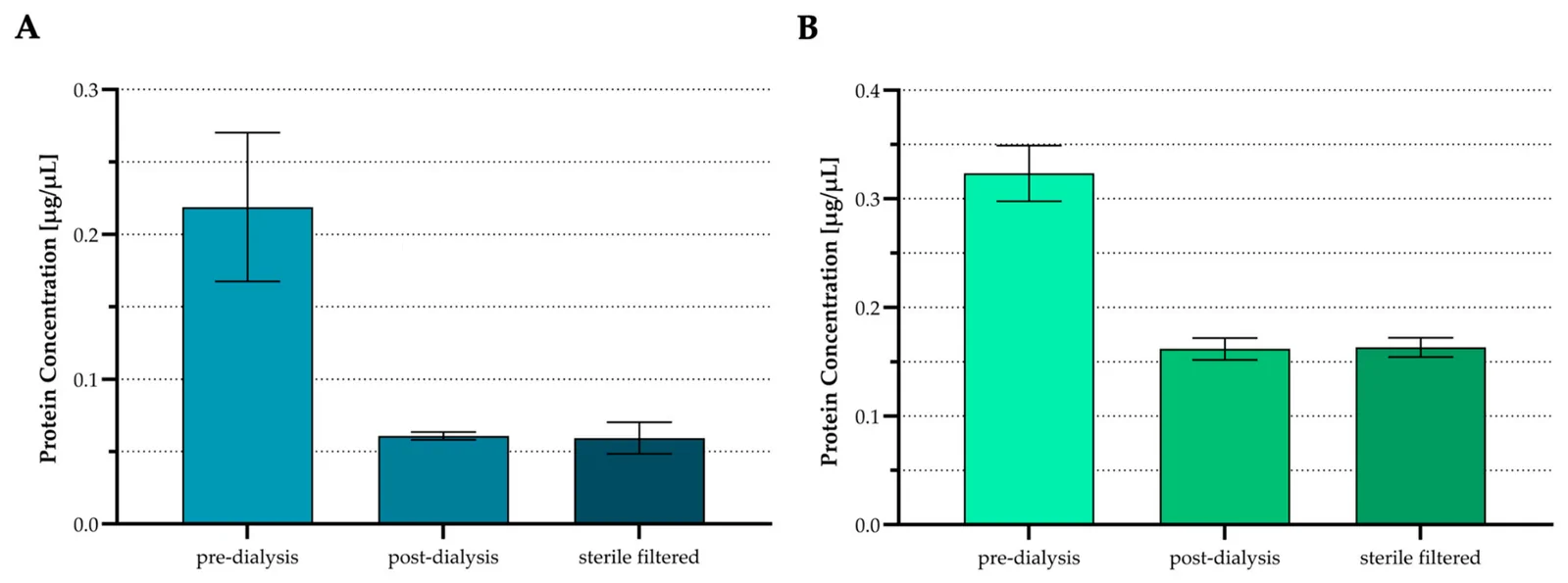
Protein concentrations during ECM extract processing. Apparent BCA-detectable protein concentrations of cortical DBG-derived ECM extracts were determined after extraction, after dialysis, and after sterile filtration. (**A**) GuHCl-derived ECM extract. (**B**) Urea-derived ECM extract. Data are presented as mean ± SD from technical triplicate BCA measurements from one extraction workflow per buffer condition. No inferential statistical testing was performed. Error bars represent variability among technical BCA replicate measurements.

ECM proteins from cortical DBG were extracted once using either GuHCl or urea, and apparent BCA-detectable protein concentrations were measured after extraction, after dialysis, and after sterile filtration. For the GuHCl-derived ECM extract, the highest mean protein concentration was measured after extraction, followed by lower mean concentrations after dialysis and after sterile filtration (Figure 1A). The mean protein concentrations after dialysis and after sterile filtration showed similar results. For the urea-derived ECM extract, the highest mean protein concentration was also measured after extraction, followed by lower mean concentrations after dialysis and after sterile filtration (Figure 1B). At all three measurement points, the urea-derived ECM preparation showed higher mean apparent BCA-detectable protein concentrations than the GuHCl-derived ECM preparation.

### 3.3. SEAP Activity and PrestoBlue™ Metabolic Activity After Stimulation with Cortical DBG-Derived ECM Extracts

HEK-Blue™ TGF-β Reporter cells were stimulated with cortical DBG-derived ECM extracts generated using either GuHCl or urea. GuHCl was used as the reference extract for defining the ECM protein input. The three treatment conditions corresponded to apparent BCA-detectable ECM protein inputs of 11.89 µg, 17.84 µg, and 23.78 µg per well, referred to as ECM^1^, ECM^2^, and ECM^3^, respectively. Urea extract volumes were adjusted to match the corresponding GuHCl-derived apparent BCA-detectable protein amounts.

**Figure 2:**
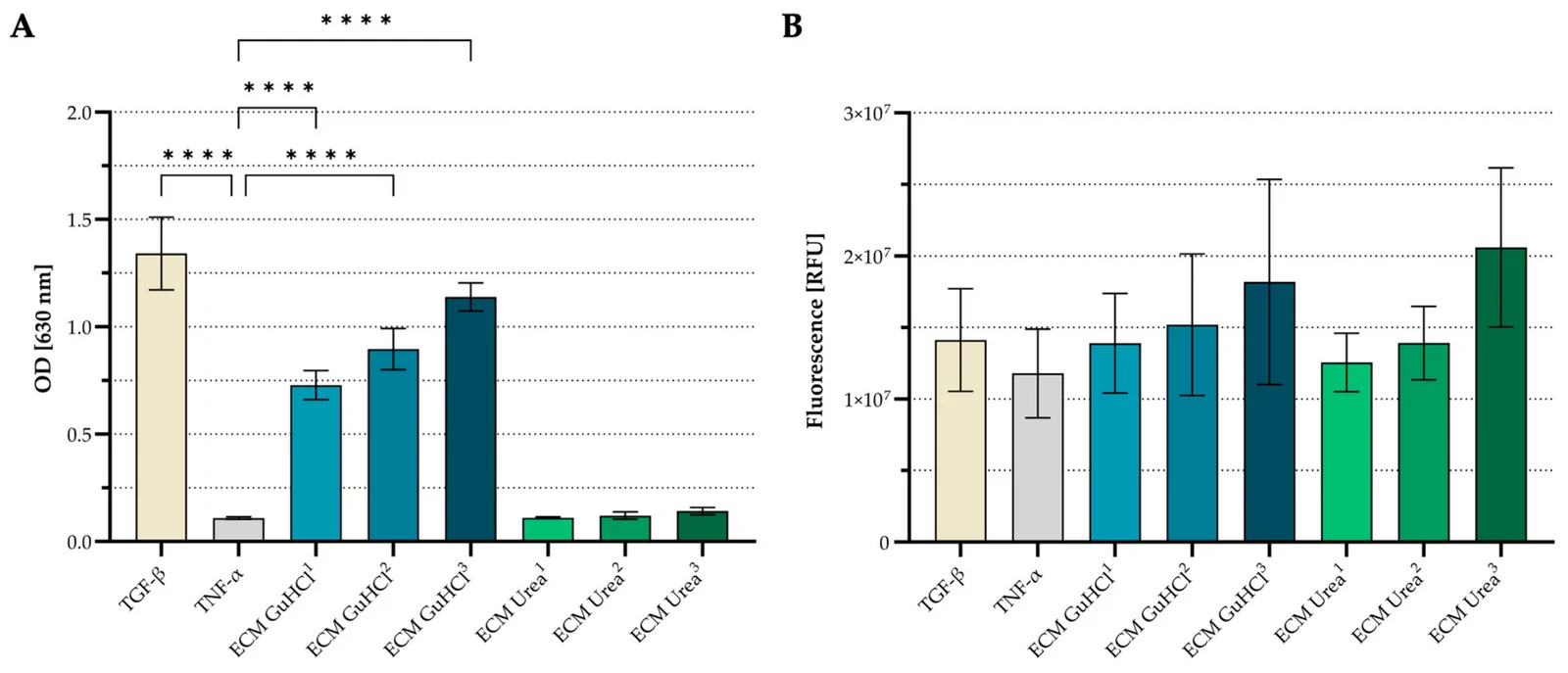
SEAP reporter activity and PrestoBlue™-based viability/metabolic activity after stimulation with cortical DBG-derived ECM extracts. HEK-Blue™ TGF-β Reporter cells were stimulated for 48 h with GuHCl- or urea-derived ECM extracts from cortical DBG. GuHCl served as a reference for defining the 20, 30, and 40 vol% conditions, corresponding to 11.89 µg, 17.84 µg, and 23.78 µg apparent BCA-detectable ECM protein per well, respectively. Urea extract volumes were adjusted to match the corresponding apparent BCA-detectable protein input. (**A**) SEAP activity measured as OD at 630 nm. (**B**) PrestoBlue™-based viability/metabolic activity measured by fluorescence. TGF-β served as a positive control and TNF-α as a pathway-negative cytokine control. Data are presented as mean ± SD from three independent cell-culture experiments after averaging technical triplicates within each experiment. Statistical analysis was performed for SEAP activity and PrestoBlue™-based viability/metabolic activity using one-way ANOVA followed by Dunnett’s multiple-comparisons test against the TNF-α control. No significant differences were observed in PrestoBlue™-based viability/metabolic activity, and these data were not used to normalize SEAP activity. Significance is indicated as follows: **** *p* ≤ 0.0001.

SEAP activity differed between GuHCl- and urea-derived ECM extract conditions (Figure 2A). The recombinant TGF-β positive-control condition showed the highest SEAP signal among the control conditions, whereas the TNF-α pathway-negative cytokine control showed low SEAP activity. GuHCl-derived ECM extracts showed increasing mean SEAP activity from GuHCl-ECM^1^ to GuHCl-ECM^3^. All GuHCl-derived ECM extract conditions were significantly higher than the TNF-α control. Urea-derived ECM extracts showed low SEAP activity across all three matched protein inputs and did not differ significantly from the TNF-α control. PrestoBlue™-based viability/metabolic activity was measured in parallel (Figure 2B). PrestoBlue™ fluorescence signals were detected across all control and ECM extract conditions. Mean fluorescence values were highest for GuHCl-ECM^3^ and Urea-ECM^3^. However, PrestoBlue™-based viability/metabolic activity data were interpreted with caution and were not used to normalize SEAP activity.

### 3.4. Direct Stimulation with Processed Cortical and Cancellous Allograft Products

**Figure 3:**
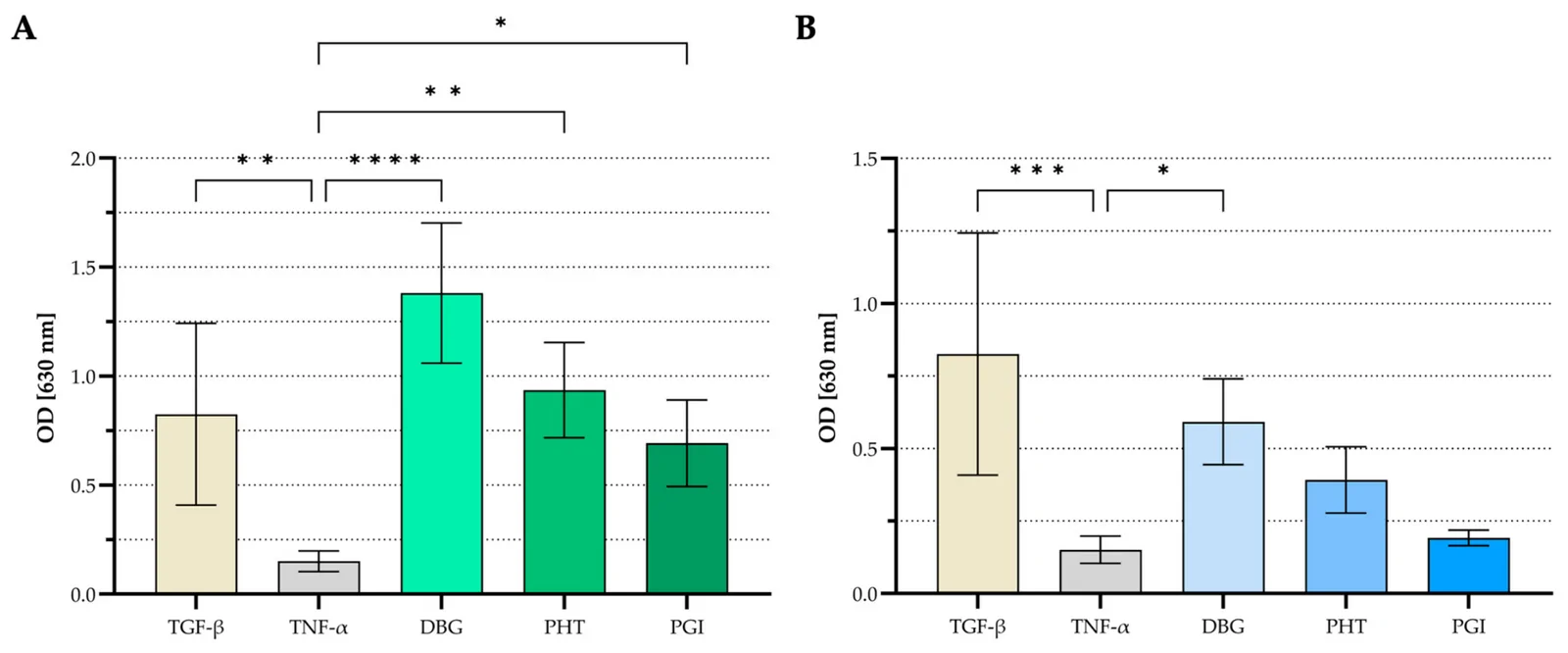
SEAP reporter activity after direct stimulation with processed cortical and cancellous allograft products. HEK-Blue™ TGF-β Reporter cells were incubated for 48 h in direct contact with DBG, PHT, or PGI allograft products. (**A**) Cortical allograft products. (**B**) Cancellous allograft products. TGF-β served as a positive control, and TNF-α as a pathway-negative cytokine control. SEAP activity was measured as OD at 630 nm. For DBG, PHT, and PGI conditions, data are presented as mean ± SD from three independent cell-culture experiments after averaging technical triplicates and subsequently averaging the three product batches within each experiment. For recombinant TGF-β and TNF-α controls, values represent the means of three independent experiments after averaging technical triplicates. Statistical analysis was performed using one-way ANOVA followed by Dunnett’s multiple-comparisons test against the TNF-α negative control. No pairwise statistical comparisons were performed between DBG, PHT, and PGI or between cortical and cancellous groups. As DBG, PHT, and PGI were applied as supplied product formats and were not normalized to dry DBM content, accessible surface area, hydration state, or cell number, direct comparisons among product formats are descriptive. Significance is indicated as follows: * *p* ≤ 0.05, ** *p* ≤ 0.01, *** *p* ≤ 0.001, **** *p* ≤ 0.0001.

HEK-Blue™ TGF-β reporter cells were cultured for 48 h in direct contact with processed cortical or cancellous allograft products, including DBG, PHT, and PGI. Cortical and cancellous products were analyzed separately. Under the applied direct-contact assay conditions, cortical DBG, PHT, and PGI each induced SEAP activity above the TNF-α pathway-negative cytokine control. As these materials were applied as supplied product formats and were not normalized to dry DBM content, accessible surface area, hydration state, or cell number, relative signal magnitudes among DBG, PHT, and PGI were interpreted descriptively (Figure 3).

Within the cortical panel (Figure 3A), the non-normalized mean SEAP signal was highest for DBG under the applied assay conditions. All three cortical material groups showed significantly higher SEAP activity than the TNF-α pathway-negative cytokine control. Among cancellous products (Figure 3B), only DBG showed significantly higher SEAP activity than the TNF-α pathway-negative cytokine control, whereas cancellous PHT and PGI did not differ significantly from the TNF-α control under the present 48 h assay conditions. These findings describe non-normalized reporter outputs under the applied direct-contact setup and do not indicate absence of broader biological activity in cancellous PHT or PGI.

Corresponding no-cell controls were included for each allograft product and product batch. In both cortical and cancellous panels, no-cell controls showed low OD values within the range of the TNF-α negative control (Supplementary Figure S1A,B). Cell-containing material conditions showed higher OD values than their corresponding no-cell controls for DBG, PHT, and PGI, except for cancellous PGI, which remained close to the no-cell and TNF-α control ranges.

PrestoBlue™ readouts were included as exploratory assay-quality control data in one of the three independent direct-stimulation cell-culture experiments. In both cortical and cancellous panels, PrestoBlue™ fluorescence signals were detected in all cell-containing material conditions (Supplementary Figure S2A,B). Mean fluorescence values for DBG, PHT, and PGI were lower than the cytokine control conditions. Because these data were obtained from only one independent experiment and material-associated diffusion barriers, physical shielding, adsorption, or optical/fluorescence interference could not be excluded, they were not interpreted as validated viability, proliferation, cytotoxicity, or cell-number endpoints and were not used to normalize SEAP activity.

### 3.5. SEAP Activity of Donor-Specific DBM Sponge Samples

**Figure 4:**
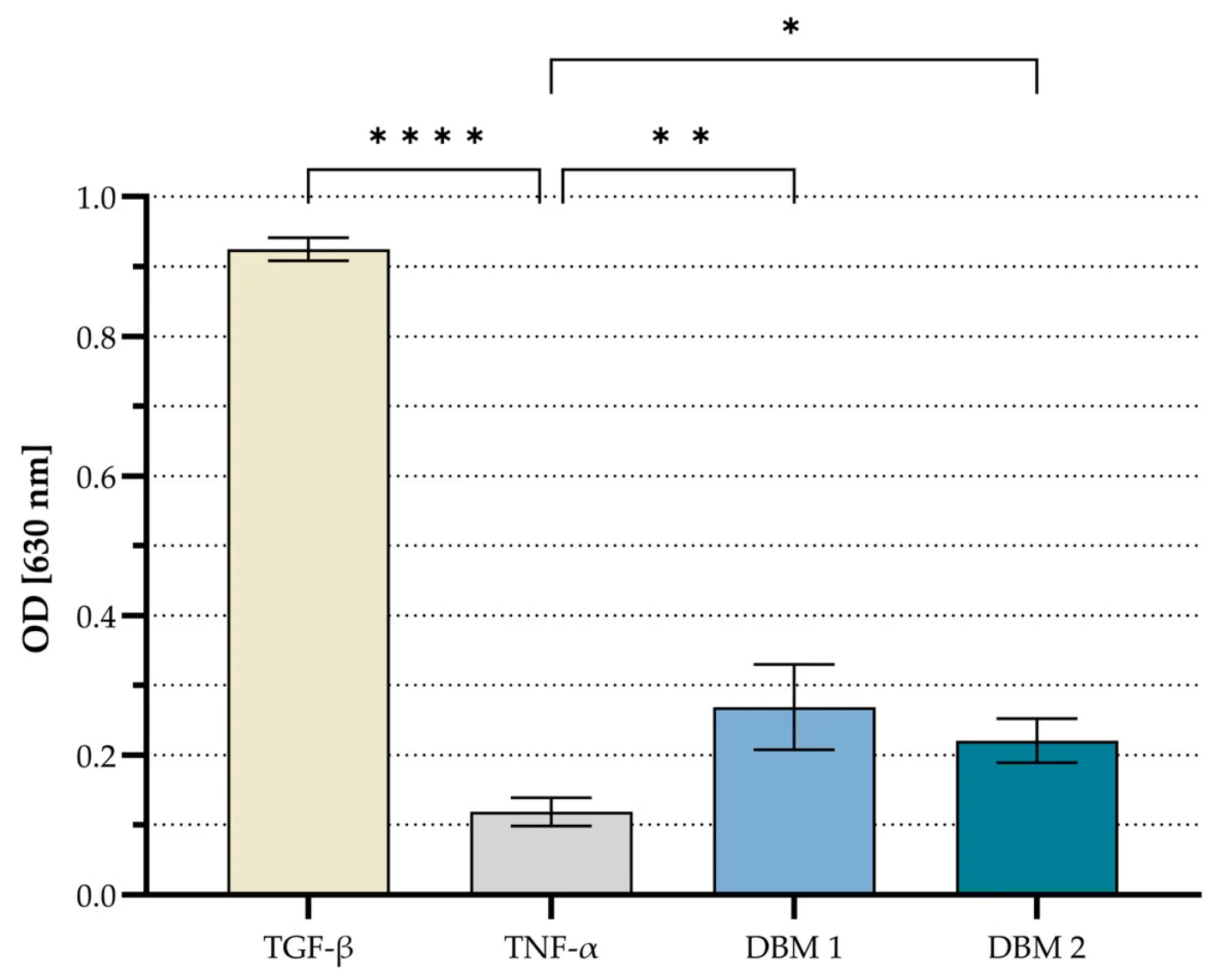
SEAP reporter activity of donor-specific DBM sponge samples. Two donor-specific DBM sponge samples, DBM 1 and DBM 2, were incubated with HEK-Blue™ TGF-β Reporter cells for 48 h. SEAP activity was measured as OD at 630 nm. TGF-β served as positive control and TNF-α as pathway-negative cytokine control. Data are presented as mean ± SD from three independent cell-culture experiments after averaging technical triplicates within each experiment. DBM 1 and DBM 2 represent two individual donor-specific sponge samples and were analyzed separately. Statistical analysis was performed using one-way ANOVA followed by Dunnett’s multiple-comparisons test against the TNF-α control. No material-only no-cell controls were included for the DBM sponge samples. Therefore, potential material-derived background cannot be fully excluded for this format. Significance is indicated as follows: * *p* ≤ 0.05, ** *p* ≤ 0.01, **** *p* ≤ 0.0001.

Two donor-specific DBM sponge samples, DBM 1 and DBM 2, were tested separately in the HEK-Blue™ TGF-β reporter assay. The recombinant TGF-β positive-control condition showed the highest mean SEAP activity among all conditions, whereas the TNF-α pathway-negative cytokine control showed the lowest mean signal (Figure 4). Both DBM sponge samples showed higher mean SEAP activity than the TNF-α control. DBM 1 showed a higher mean SEAP signal than DBM 2. Both DBM 1 and DBM 2 were significantly different from the TNF-α control. Statistical testing reflects reproducibility of the reporter response across independent cell-culture experiments for each individual sponge sample and does not represent donor-population variability. As material-only no-cell controls were not included for the DBM sponge format, potential material-derived QUANTI-Blue™ background or interference cannot be fully excluded. Therefore, the DBM sponge data were interpreted as feasibility data for applying the reporter workflow to structured DBM samples, not as validated quantitative donor-variability or product-performance data.

## 4. Discussion

### 4.1. Assay Scope and Overall Interpretation

This study evaluated the HEK-Blue™ TGF-β Reporter Assay as a targeted functional readout for Smad-dependent reporter activation in DBM-derived allograft materials. The assay was not intended to quantify active TGF-β protein concentration or to rank overall graft quality, but to detect Smad-dependent reporter activation under standardized in vitro conditions. The results show that several DBM-derived material conditions induced measurable SEAP activity in a Smad-inducible HEK-Blue™ TGF-β reporter system. As the reporter cells express SEAP under the control of a Smad-inducible promoter, the assay provides a targeted TGF-β/Smad reporter readout rather than a nonspecific metabolic or inflammatory readout. Recombinant TGF-β confirmed assay responsiveness, whereas TNF-α served as the pathway-negative cytokine control. Nevertheless, pathway inhibition or TGF-β neutralization was not included. Therefore, the present study does not provide pharmacological receptor-blocking confirmation of the DBM-induced reporter response.

### 4.2. ECM Extraction, Protein Recovery, and Functional Activity

The extraction-based experiments showed that biochemical protein recovery, as assessed by apparent BCA-detectable protein concentration, did not predict reporter activation in the preparations tested. The urea-derived extract contained a higher apparent BCA-detectable protein concentration than the GuHCl-derived one, yet only the GuHCl-derived extract induced increasing SEAP activity at matched apparent BCA-detectable protein input. This supports the interpretation that biochemical protein recovery and pathway-active recovery are not equivalent endpoints. GuHCl may more effectively disrupt extracellular matrix interactions and release matrix-bound growth-factor complexes or associated regulatory proteins relevant to TGF-β/Smad reporter activation. In contrast, urea may solubilize abundant ECM proteins that are not active in this reporter assay. In addition, urea extraction can affect protein conformation and, under certain conditions, may promote chemical modifications such as carbamylation, which can affect protein function [43]. However, these mechanisms were not directly tested here. Extraction-based testing is useful as it allows defined dosing, comparison at matched apparent BCA-detectable protein input, and compatibility with soluble cell-based assays. Protein extraction can also change the biological state of matrix-associated factors by disrupting protein–matrix interactions, altering ligand accessibility, dissociating protein complexes, or changing protein conformation [22,23,24,25,26,27,37,44]. Although dialysis was performed to support cell compatibility and may have removed low-molecular-weight components below the 1 kDa MWCO, changes in apparent protein concentration after dialysis most likely reflect changes in sample volume during dialysis rather than loss of intact proteins alone [27]. Therefore, extraction should be interpreted as a controlled screening format rather than a direct representation of the native material state. These findings support the use of functional reporter assays as a complement to protein quantification, particularly as immunochemical or biochemical detection of growth-factor-related components does not necessarily demonstrate pathway activation [18].

### 4.3. Direct Product Testing and Interpretation of Cortical Versus Cancellous Responses

Direct product testing assessed whether processed allograft materials induced reporter activation in their supplied-material state. This format is relevant because DBM-derived products are used clinically as structured materials rather than as purified soluble extracts. Direct stimulation preserved material geometry, hydration, matrix-bound components, and surface-associated cues, but also introduced assay-specific variables, including diffusion, cell distribution, local cell–material contact, and adsorption of soluble assay components [28,29].

Cortical DBG, PHT, and PGI all induced significantly higher SEAP activity than the TNF-α pathway-negative control. In both the cortical and cancellous panels, DBG showed the highest mean non-normalized reporter output, followed by PHT and PGI. This consistent pattern supports a qualitative ranking of the observed mean non-normalized reporter outputs among the investigated product conditions under the applied assay conditions. Heat treatment, rehydration and Putty formulation, and gamma irradiation can affect protein integrity, conformation, matrix interactions, and the accessibility or release of matrix-associated signaling components [9,10,11,12,13,14,15,16]. Together with prior proteomic characterization showing retention of structural and matrix-organization-associated ECM protein signatures in processed cortical allografts, the present findings demonstrate that functionally detectable reporter activity is present in cortical PHT and PGI despite heat treatment, Putty formulation, and subsequent gamma irradiation [45].

The observed order should be interpreted as a qualitative ranking of non-normalized reporter outputs rather than as a quantitative measurement of the effect attributable to each individual processing step. Because DBG, PHT, and PGI were tested as supplied product formats and were not normalized to dry DBM content, accessible matrix surface, hydration state, or cell number, the magnitude of processing-related changes cannot be quantified. In addition, as PHT and PGI were evaluated as complete supplied formulations, potential carrier-related effects cannot be separated from effects associated with the DBM fraction. Nevertheless, the consistent ordering observed in both material groups, together with significant reporter activation by cortical PHT and PGI, supports the ability of the assay to distinguish qualitative differences associated with the investigated processing states while confirming functional reporter activity in processed products. TGF-β signaling is strongly regulated by matrix association, latent-complex formation, release kinetics, and activation state, which may contribute to the observed differences in reporter output [30,31,32,33,34,35,36,46].

Among cancellous products, only DBG showed significantly higher SEAP activity than the TNF-α control. Cancellous PHT and PGI showed lower mean non-normalized reporter outputs than DBG but did not differ significantly from the TNF-α control. This finding is consistent with the qualitative ordering observed in both material groups under the applied 48 h assay conditions but does not indicate that cancellous materials are biologically inferior. Cancellous bone differs from cortical bone in architecture, matrix density, marrow association, surface area, and remodeling environment, which may influence how matrix-associated factors are retained, released, or presented after processing. Previous proteomic characterization showed that cancellous-derived allografts may be relatively enriched in proteins associated with inflammatory, coagulative, and immune-related processes, whereas cortical-derived grafts showed higher abundance of structural matrix proteins [45]. Thus, cancellous materials may retain biologically relevant activities related to early remodeling, immune modulation, coagulation, fibrovascular ingrowth, or cell recruitment that are not fully captured by a TGF-β/Smad reporter assay [45,47]. Moreover, longer material–cell exposure times may increase the detectability of slowly released or weakly presented reporter-active components, while extended QUANTI-Blue™ development within the validated detection window may further influence analytical sensitivity. Therefore, the absence of significant reporter activation for cancellous PHT and PGI in the present 48 h setup should be interpreted as assay-condition dependent and does not exclude reporter activation under extended assay conditions.

### 4.4. Assay Controls, Material Interference, and Donor-Specific DBM Sponges

No-cell controls were essential for interpreting direct product stimulation and are shown in Supplementary Figure S1. Their low OD values, generally within the range of the TNF-α control, indicate that the tested DBG, PHT, and PGI materials did not generate a substantial material-derived background signal. The higher OD values in cell-containing material conditions therefore support a cell-dependent reporter response rather than direct material-associated color development. In contrast, PrestoBlue™ metabolic readouts after direct material stimulation were interpreted descriptively and are shown in Supplementary Figure S2. Resazurin-based assays depend on the diffusion of the reagent and the cellular reduction of resazurin to fluorescent resorufin. In scaffold-like or material-containing systems, the readout can be affected by incubation time, reagent depletion, cell density, diffusion barriers, adsorption, and optical or fluorescence interference [48,49]. In the present direct-contact setup, solid particles and hydrated Putty formulations may have physically shielded cells from homogeneous reagent exposure or reduced effective redox conversion within the local material-associated microenvironment. Therefore, lower PrestoBlue™ signals in response to direct material stimulation should not be interpreted as reduced viability per se. For the same reason, PrestoBlue™ was not used to normalize SEAP activity in the direct-contact material experiments. In this setup, cells and materials were physically co-localized, and independent recovery of cells from particulate or Putty-like materials would introduce additional detachment and recovery bias. Direct cell counting would require separate validation for each material format, particularly as DBM-derived materials may contribute material-associated background or interfere with cell recovery. Development and validation of a material-compatible normalization strategy therefore remain separate methodological requirements for future assay optimization. Accordingly, the present study reports direct-contact SEAP activity as a non-normalized reporter output, supported by material-only no-cell QUANTI-Blue™ controls for DBG, PHT, and PGI.

The donor-specific DBM sponge experiments showed that the workflow can generate detectable SEAP responses in structured, non-homogenized DBM-derived samples. Both sponge samples showed SEAP activity above the TNF-α control, supporting the feasibility of applying the reporter workflow beyond granules and Putty formulations. The different signal intensities may reflect sample-specific differences in retained matrix-associated activity, structure-dependent diffusion, or local cell–material contact. As only two sponge samples were tested and no no-cell controls were included for this format, these data do not allow conclusions about donor variability, product-class performance, or clinical efficacy. Accordingly, the sponge results should be considered feasibility data demonstrating that the workflow can be applied to structured DBM samples.

## 5. Conclusions

Overall, the HEK-Blue™ TGF-β Reporter Assay provides a practical and rapid functional readout for Smad-dependent TGF-β reporter activation in DBM-derived allograft materials. The assay can complement biochemical protein measurements by indicating whether extracted or directly applied DBM-derived materials induce reporter activation under defined in vitro conditions. In its current form, the assay is best interpreted as an exploratory, pathway-oriented screening tool. Quantitative comparison of processing stages, product formats, or batches will require further analytical validation, including receptor-level pathway inhibition or neutralization, development and validation of a material-compatible cell-number normalization strategy, normalization to dry DBM content or accessible matrix surface, and consideration of product-format-related effects, including hydration state and geometry. Future studies could combine this reporter assay with complementary osteogenic, angiogenic, immune-modulatory, and matrix-remodeling assays to further characterize the multifactorial biology of DBM-mediated bone repair.

## Data Availability

The raw data supporting the findings of this study are provided as Supplementary Data files. These files include the raw absorbance and fluorescence values, technical replicate data, anonymized intermediate measurements where applicable, averaged experiment-level values, and statistical analysis outputs underlying Figure 1, Figure 2, Figure 3 and Figure 4 and Supplementary Figures S1 and S2. No identifiable donor-level data are included, as the study was performed using pooled, processed, and irreversibly anonymized allograft material or donor-specific DBM sponge samples without access to identifiable donor information.

## Supplementary Materials

The following supporting information can be downloaded at: https://www.mdpi.com/article/10.3390/jfb17080416/s1. File S1: Data. Raw absorbance and fluorescence values, technical replicate data, averaged experiment-level values, anonymized intermediate measurements where applicable, and statistical outputs underlying Figure 1, Figure 2, Figure 3 and Figure 4 and Supplementary Figures S1 and S2. Figure S1: SEAP reporter activity after direct stimulation with processed cortical and cancellous allograft products and corresponding material-only no-cell controls; Figure S2: Exploratory PrestoBlue™ metabolic activity readouts after direct stimulation with processed allograft products.

## Abbreviations

The following abbreviations are used in this manuscript:

ABC	ammonium bicarbonate
BCA	bicinchoninic acid
DBG	demineralized bone granules
DBM	demineralized bone matrix
ECM	extracellular matrix
GuHCl	guanidine hydrochloride
OD	optical density
PBS	phosphate-buffered saline
PGI	gamma-irradiated PHT
PHT	wet heat-treated DBG formulated as Putty
RFU	relative fluorescence units
SEAP	secreted embryonic alkaline phosphatase
TGF-β	transforming growth factor beta
TNF-α	tumor necrosis factor alpha
